# Intraday and Interday Evaluation of pH and Hydrogen Peroxide in the Exhaled Breath Condensate of Horses Using A Portable Device

**DOI:** 10.1021/acsomega.5c05941

**Published:** 2025-10-06

**Authors:** Bianca Barbosa, Thasla F. Santi, Ana C. Rodak, Maria F. Nogara, Lidiane M. B. Leite, Saulo H. Weber, Cleber Niels, Ruan R. Daros, Pedro V. Michelotto

**Affiliations:** † Graduate Program in Animal Science, Pontifícia Universidade Católica Paraná, Rua Imaculada Conceição 1155, Prado Velho, 80215-901 Curitiba, PR, Brazil; ‡ Veterinary Medicine, Pontifícia Universidade Católica Paraná, Rua Imaculada Conceição 1155, Prado Velho, 80215-901 Curitiba, PR, Brazil; § Core for Cell Technology, School of Medicine and Life Sciences, 28100 Pontifícia Universidade Católica do Paraná, Rua Imaculada Conceição 1155, Prado Velho, 80215-901 Curitiba, PR, Brazil; ∥ EthoLab − Applied Ethology and Animal Welfare Lab, Pontifícia Universidade Católica do Paraná, Rua Imaculada Conceição 1155, Prado Velho, 80215-901 Curitiba, PR, Brazil

## Abstract

The analysis of equine exhaled breath condensate (EBC) lacks standardized methodology, and current collection devices are often adapted for research. This study evaluates a novel horse-specific EBC collector and assesses the variability of EBC pH and hydrogen peroxide (H_2_O_2_) levels, exploring potential correlations with bronchoalveolar lavage (BAL) and tracheal wash (TW) cytology. Eleven healthy mixed-breed mares from a teaching herd, with no evidence of airway abnormalities, were included in this randomized observational study. The collection efficiency of the proposed device was assessed, and intra- and interday variations in EBC pH and H_2_O_2_ levels were analyzed. Airway endoscopy, tracheal mucus scoring, and TW and BAL fluid cytology were also performed. EBC pH showed no significant intra- (*P* = 0.631, ES 0.008–0.456) or interday (*P* = 0.864, ES 0.116–0.365) variation, nor did H_2_O_2_ levels (*P* = 0.953, ES 0.077–0.185; *P* = 0.929, ES 0.019–0.190, respectively). In this study, no correlations were found between EBC parameters and BAL or TW cytology. However, 34.5% of pH samples and 32.7% of H_2_O_2_ samples were insufficient for analysis due to low sample volume. These findings suggest that EBC collection using the horse-specific device is feasible and that pH and H_2_O_2_ levels remain stable regardless of collection time. However, further refinement of the device is necessary to improve sample yield and ensure reliable analysis.

## Graphical abstract

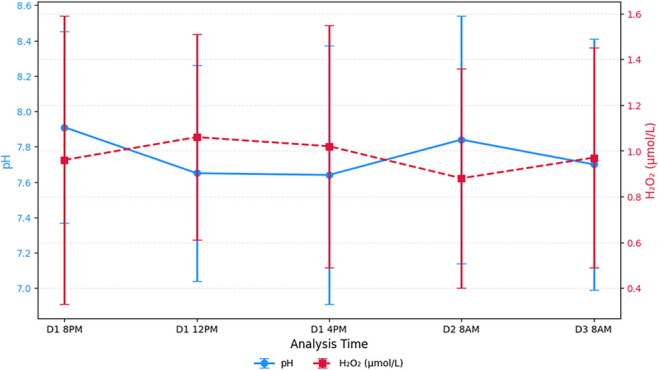

## 1 Introduction

The collection of exhaled breath condensate (EBC) is a noninvasive method for obtaining samples that represent the fluid lining the surface of the airways. Consequently, EBC samples can be used to identify diagnostic biomarkers for respiratory diseases in various animal species.^1–3^ Among the primary biomarkers identified so far are hydrogen peroxide (H_2_O_2_) and pH.^1,4,5^ In horses, acidification of EBC pH has been associated with mild to moderate equine asthma,^1^ and elevated pH values have been correlated with airway inflammation and neutrophil percentage in bronchoalveolar lavage (BAL) fluid.^5^ Hydrogen peroxide, a reactive oxygen species produced mainly by activated inflammatory cells during oxidative stress, is considered a valuable biomarker for monitoring respiratory disease severity.^6,7^ In horses, EBC H_2_O_2_ concentrations have been positively correlated with neutrophil counts in BAL fluid.^5^

Several studies in humans and equines have reported significant diurnal variation in EBC biomarkers, including H_2_O_2_ and pH, suggesting that time of collection may influence biomarker levels.^8–10^ For example, Nowak et al.^8^ demonstrated circadian rhythms in exhaled H_2_O_2_ in healthy human volunteers, and van Beurden et al.^9^ found increasing H_2_O_2_ concentrations throughout the day in both healthy individuals and patients with chronic obstructive pulmonary disease. Similarly, Duz et al.^1^ reported diurnal variation of EBC H_2_O_2_ and pH in horses. However, some equine studies have found no significant diurnal changes in these biomarkers,^10^ reflecting variability in findings that may be influenced by methodological differences.

Currently, analyses of EBC lack standardized collection and measurement protocols, and equipment used for horses are often adapted from human devices or designed for research purposes, limiting routine clinical application.^11^ Therefore, the development and validation of horse-specific, portable EBC collection devices are imperative for reliable biomarker measurement and clinical monitoring.

In this study, we introduce a potential portable device specifically designed for equine EBC collection. Using this equipment, we aimed to initially evaluate its sampling capacity and assess intra- and interday variability of EBC pH and hydrogen peroxide (H_2_O_2_) levels in horses classified as healthy based on historical records and physical examination. Additionally, we investigated potential correlations between these EBC parameters and cytological findings from tracheal wash (TW) and bronchoalveolar lavage (BAL) fluid.

## 2 Material and Methods

### 2.1 Ethical Approval

Ethical approval was obtained from the Ethics Committee on Animal Use of the Pontifical Catholic University of Paraná (PUCPR), under protocol number 02259 (August 2022).

### 2.2 Study Design

Eleven mixed-breed mares, aged between 4 and 13 years, from the herd of the Experimental Farm of PUCPR, were used in the study. All animals underwent a clinical examination to ensure the absence of any abnormal conditions.

Subsequently, the mares were randomly evaluated during the study period. The EBC assessments were conducted both intraday and interday. On the first day, collections occurred at 8 AM, 12 PM, and 4 PM, while on the second and third days, collections took place at 8 AM, adapted from a previously described study.^1^ During the EBC collections, ambient temperature and relative humidity were recorded using a thermo-hygrometer (Incoterm, São Paulo, Brazil), and respiratory and heart rates were assessed using a stethoscope. On the day following the last EBC collection, airway endoscopy was performed to evaluate tracheal mucus scores, along with TW and BAL fluid collections from each animal.

### 2.3 Equipment for EBC Collection

The horse EBC collector, developed by our research group, was fabricated using polylactic acid (PLA) in a 3D printer (CL2 Pro Plus-Cliever, Belo Horizonte, Brazil) at PUCPR’s machining facility.

To operate the device, a plastic zip-lock bag containing ice is first placed in the cooling chamber, and the collector is then completely wrapped in an A4 plastic sheet (Figure 1). This setup allows the warm exhaled air to encounter the cold wall of the cooling chamber, causing condensation.

**Figure 1:**
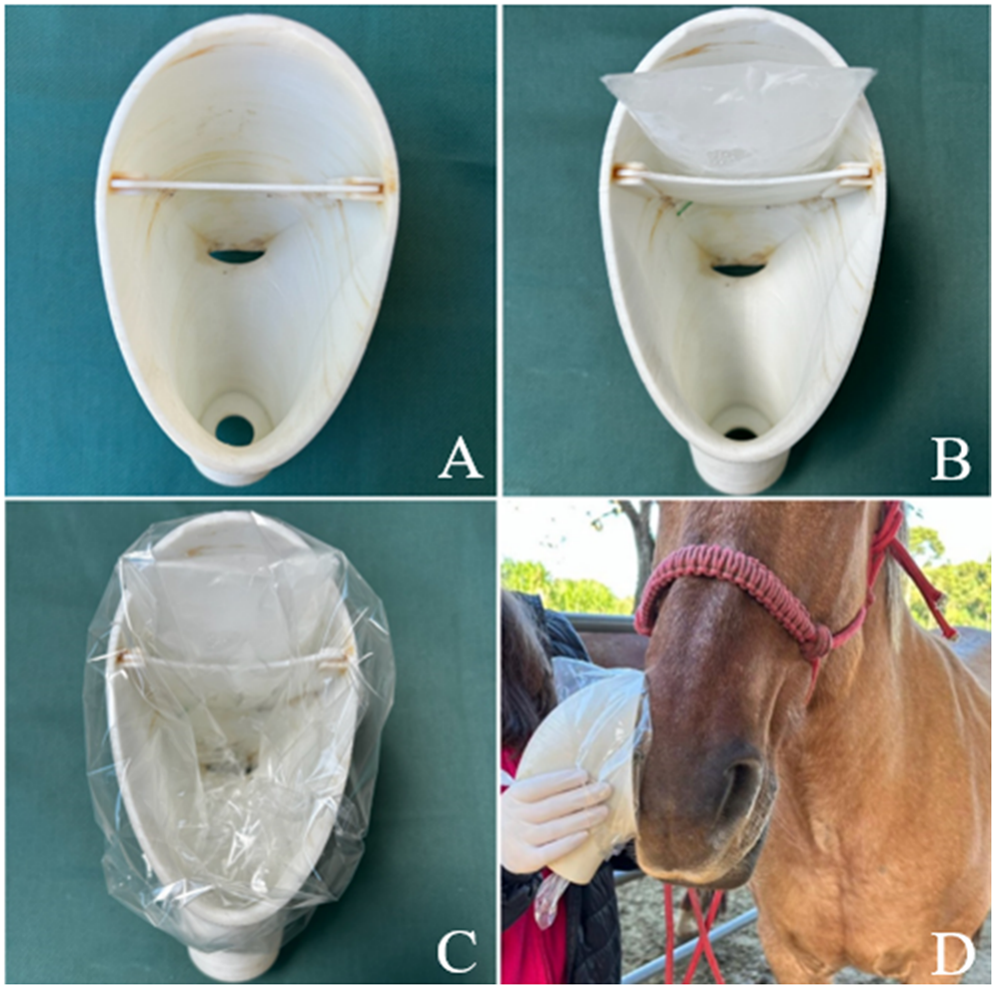
(A) Front view of the equine EBC collector. The top part is the cooling chamber (approximately 4.5 cm in height and 7.5 cm in width), and the bottom part is the condensation chamber (approximately 9 cm in height and 8.5 cm in width). (B) EBC collector cooling chamber filled with ice. (C) EBC collector wrapped in a plastic bag, ready for use, and allows the warm exhaled air to encounter the cold wall of the cooling chamber, causing condensation. (D) Collector of EBC positioned in the horse’s nostril.

The nostrils of horses were first cleaned using dry gauzes. The equipment was then positioned over one of the horse’s nostrils for 15 min. After this period, the liquid sample collected inside the plastic bag was extracted using an automatic micropipette, then stored in cryotubes. The samples were immediately cooled in a styrofoam container with ice and transferred to storage in liquid nitrogen at −196 °C, where they were maintained until laboratory analysis.

The methodology employed in this study, particularly the air-cooling technique, nostril cleaning and duration of collect, was based on pilot studies conducted prior to the start of this research,^12^ and studies by other authors, carried out previously.^4,14,20,22,23^

### 2.4 EBC Processing and Analysis

Within 30 days of collection, the samples were removed from liquid nitrogen and analyzed. Prior to analysis, they were thawed at room temperature (approximately 20 °C) for about 15 min, until completely thawed. All analyses were performed on the same day and at the same time to ensure consistency.

EBC pH was measured using a pocket pH meter (HI98103, Hanna Instruments, São Paulo, Brazil). A minimum sample volume of 100 μL was required to fully submerge the electrode bulb. Hydrogen peroxide (H_2_O_2_) concentration in EBC samples was determined via spectrophotometry (VersaMax Microplate Reader, Molecular Devices, San Jose, California) using the peroxidase-based Amplex Red Hydrogen Peroxide/Peroxidase Assay Kit (Molecular Probes Inc., Eugene, Oregon), following the manufacturer’s instructions. Each analysis required 50 μL of sample. Absorbance was measured at 560 nm, and results are expressed as μmol/L.

### 2.5 Airways Endoscopy, TW and BAL Collection, Processing and Analysis

Airway endoscopy and the collection of TW and BAL samples from each mare were performed on the day following the last EBC collections. The amount of mucus in the tracheal lumen was evaluated using a scoring system ranging from 0 to 5. Additionally, TW samples were collected using 20 mL of sterile saline, and BAL fluid was collected using 300 mL of sterile saline, as previously described.

All collected materials were placed in a sterile siliconized plastic container and refrigerated until sample processing, which occurred within a maximum of 1 h after collection. After processing, slides were prepared for cytological analysis and stained with Romanowsky stain (Panótico Rápido, Laborclin, Pinhais, Brazil). For the cytological analysis, 300 cells were counted.

For TW, cytological analysis was considered normal when it showed less than 20% neutrophils, 10% lymphocytes, and less than 1% eosinophils [10]. For BAL, normal values were defined as less than 5% neutrophils, up to 1% eosinophils, and 2% mast cells.^11^

### 2.6 Statistical Analysis

Initially, a descriptive and qualitative analysis was conducted, along with the comparison of means. The normality of all data was tested using the D’Agostino-Pearson test. The paired *t* test was applied for dependent samples and continuous variables, which increased statistical power despite sample loss related to the technique. The Pearson correlation test was used to correlate the obtained data, with *P*-values less than 0.05 considered significant. Results are presented as means, standard deviations, and 95% confidence intervals. Effect size (ES) was assessed using Cohen’s d for the comparison of means and Pearson’s r for correlations to quantify the magnitude of observed effects independent of sample size, providing a measure of practical or biological relevance.

## 3 Results

Regarding the collection procedures, all animals tolerated both the equipment and the process well. While initial reactions were observed, the animals adapted to the equipment as the collections progressed. However, this adaptation was not systematically investigated in the present study. There were instances of sample loss due to minimal or insufficient volume, which sometimes rendered the samples inadequate for pH and H_2_O_2_ analysis.

Out of the five planned samples for each animal, totaling 55 samples, only two collections (3.6%) yielded no volume. Since the pH measurement required a minimum volume of 100 μL for the bulb of the equipment to be submerged in the sample, pH analysis was possible in 36 samples (65.5%).

For H_2_O_2_ measurement, single, duplicate, or triplicate measurements were possible for three, four, and 30 samples, respectively, considering that 50 μL of sample was required for each analysis.

### 3.1 Sample Volumes

The volume of EBC samples showed weak positive correlations with respiratory rate (*P* = 0.044, *r* = 0.272) and environmental temperature (*P* = 0.009, *r* = 0.349), as well as a weak negative correlation with relative humidity (*P* = 0.029, *r* = −0.294).

### 3.2 EBC pH and H_2_O_2_

The mean pH value and H_2_O_2_ concentration in EBC samples did not change in the intraday analysis (*P* = 0.631, ES 0.008–0.456; *P* = 0.953, ES 0.077–0.185, respectively) or the interday analysis (*P* = 0.864, ES 0.116–0.365; *P* = 0.929, ES 0.019–0.190, respectively) (Table 1).

**Table 1:** Mean ± Standard Deviation and 95% Confidence Intervals of Environmental and Animal Data during EBC Collections

	ambient		animal		EBC		
analyses	temp. (°C)	RH (%)	HR (bpm)	RR (mpm)	pH	H_2_O_2_ (μmol/L)	sample volume (μL)
D1 8PM	17.50 ± 2.15	75.00 ± 15.89	36.00 ± 7.40	17.10 ± 6.50	7.91 ± 0.54	0.96 ± 0.63	545.45 ± 524.16
	(16.29–18.63)	(73.65–76.35)	(34.74–37.26)	(15.84–18.34)	(6.91–8.91)	(0.00^1^-1.97)	(543.59–547.31)
D1 12PM	20.80 ± 2.46	69.40 ± 24.08	26.90 ± 9.30	23.50 ± 9.90	7.65 ± 0.61	1.06 ± 0.45	281.81 ± 298.35
	(19.62–21.92)	(67.96–70.76)	(25–61–28.19)	(22.15–24.75)	(6.64–8.66)	(0.08–2.04)	(280.04–283.58)
D1 4PM	21.30 ± 3.43	60.50 ± 33.05	33.10 ± 4.40	17.80 ± 6.30	7.64 ± 0.73	1.02 ± 0.53	546.36 ± 728.79
	(20.12–22.48)	(59.00–61.90)	(31.89–34.29)	(16.56–19.06)	(6.61–8.67)	(0.02–2.02)	(544.44–548.28)
D2 8AM	15.90 ± 2.04	72.50 ± 12.01	36.00 ± 4.00	17.50 ± 5.10	7.84 ± 0.70	0.88 ± 0.48	353.63 ± 369.87
	(14.82–17.08)	(71.13–73.77)	(34.81–37.19)	(16.23–18.67)	(6.82–8.86)	(0.00^1^–1.87)	(351.82–355.44)
D3 8AM	13.90 ± 3.14	56.80 ± 4.97	34.50 ± 3.30	14.20 ± 4.50	7.70 ± 0.71	0.97 ± 0.48	312.27 ± 575.47
	(12.70–15.04)	(55.59–58.03)	(33.37–35.71)	(12.97–15.39)	(6.68–8.72)	(0.00^1^–1.96)	(310.39–314.45)

^*a*^ Negative confidence interval lower limits. RH (%) – relative humidity (%). HR (bpm) – heart rate (beats per minute). RR – respiratory rate (movements per minute).

A strong positive correlation was observed between EBC pH and ambient temperature at 8 AM on the second day (D2) (*P* = 0.036, *r* = 0.797), and a strong negative correlation with relative humidity at 4 PM on day 1 of collection (*P* = 0.017, *r* = −0.762) and at 8 AM on day 2 (*P* = 0.012, *r* = −0.864).

### 3.3 Airways Endoscopy, TW, BAL and Correlations

Based on bronchoalveolar lavage (BAL) fluid cytology, the study sample included animals with both healthy cytological profiles and profiles compatible with mild to moderate asthma. Results related to BAL fluid and tracheal wash (TW) cytology, along with corresponding pH and H_2_O_2_ values at each collection time, are presented in Supplementary Tables 1–4.

Regarding TW cytology, seven horses exhibited normal profiles, while four showed inflammatory profiles (Supplementary Tables 3 and 4). Specifically, the percentage of macrophages was significantly higher in healthy horses compared to those with inflammatory profiles (63.0 ± 8.8% vs 29.1 ± 19.3%, *P* = 0.018), whereas neutrophil percentages were significantly elevated in horses with inflammatory TW profiles compared to healthy counterparts (39.1 ± 12.5% vs 7.9 ± 8.1%, *P* = 0.006). There was no significant differences in pH (*P* = 0.595) or H_2_O_2_ concentrations (*P* = 0.308) between horses with normal and inflammatory cytological profiles.

Regarding BAL fluid cytology, five horses presented normal profiles, while six displayed cytological findings consistent with asthma (Supplementary Tables 1 and 2). Similar to TW findings, macrophage percentages in BAL fluid were significantly higher in healthy horses compared to those with asthmatic profiles (60.7 ± 14.6% vs 37.4 ± 13.4%, *P* = 0.022), whereas neutrophil percentages were significantly elevated in horses with asthma compared to healthy counterparts (19.0 ± 9.4% vs 1.5 ± 0.87%, *P* = 0.003). Again, no significant differences were found in EBC pH (*P* = 0.176) or H_2_O_2_ concentrations (*P* = 0.165).

When comparing EBC parameters with BAL and TW cytological findings, only a few significant correlations were identified among the seven horses with normal TW cytology, and only at specific EBC collection time points. The percentage of neutrophils showed a strong negative correlation with pH (*P* = 0.044, *r* = −0.889), and pH and H_2_O_2_ concentrations were also negatively correlated (*P* = 0.039, *r* = −0.998). No additional significant correlations were observed between EBC variables and cytological profiles.

## 4 Discussion

This study proposes a horse-specific exhaled breath condensate (EBC) collection device designed to be portable, user-friendly, and well-tolerated by horses for respiratory disease assessment. This innovation could facilitate broader adoption of EBC analysis in equine diagnostics, as current collection methods are often invasive and may require sedation, limiting their routine use.^13^ Unlike previous studies that utilize adapted human collectors or improvised methods, this work employs a horse-specific collector designed and optimized for the species, with the aim of jointly defining and validating the technique along with the device. The EBC is advantageous because it allows for noninvasive, repeated evaluation of pulmonary status without inducing alterations. In contrast, bronchial lavage techniques, such as BAL require sedation, can increase local neutrophil counts for at least 48 h postprocedure, and involve airway lavage methods (BAL and TW), which may lead to sample dilution.

To understand the variability in EBC sample evaluations, we based our study protocol on previously described research,^1^ which also investigated pH and H_2_O_2_ levels in EBC intra- and interday. Similar to the findings of the aforementioned study,^1^ the mean pH and H_2_O_2_ levels in our study did not vary between investigation time points, suggesting that EBC sample collection can be performed at any time for diagnostic purposes. This contrasts with several human and equine studies reporting significant diurnal variation in exhaled H_2_O_2_ and pH, demonstrating circadian rhythms in EBC H_2_O_2_ in healthy human subjects, with peak values observed midday and at midnight,^8^ and increasing exhaled H_2_O_2_ levels throughout the day in both patients with chronic obstructive pulmonary disease and matched healthy controls.^9^ In horses, Duz et al.^1^ also described diurnal variation in EBC biomarkers. However, other equine studies, such as du Preez et al.^10^ found no significant diurnal changes, underscoring variability in the literature that may be attributable to differences in study design, populations, and collection methodologies.

The overall mean pH value of 7.74, as well as the values observed at different time points in this study, were higher than those reported in horses without respiratory diseases (4.30–4.63 and 5.86–6.72).^1,4^ This difference may be attributed to the varying methodologies employed, which complicates direct comparisons between studies. Additionally, some of the animals examined exhibited inflammatory cytology, which may have influenced the results. The concentrations of H_2_O_2_ described in healthy horses are reported as 0.4 μmol/L^14^ and 0.41–1.59 μmol/L,^15^ which are similar to those found in the present study, where the overall mean was 0.97 μmol/L.

Despite the stability of H_2_O_2_ values across the investigated time points, no consistent correlations were observed between H_2_O_2_ and the other variables analyzed. All animals in the study were clinically healthy and showed no evident signs of respiratory abnormalities. However, some horses presented increased inflammatory cells in TW and BAL fluid, suggesting mild to moderate equine asthma—a condition that can occur without overt clinical signs, even in horses kept under pasture conditions.^16,17^ When comparing data between horses with normal and inflammatory cytology, no significant differences were found. This suggests that a larger sample size may be necessary in future studies to better detect potential associations. While previous research has reported a correlation between elevated H_2_O_2_ levels in EBC and neutrophilic inflammation in BAL fluid,^10,18^ other studies have shown conflicting results. For instance, one investigation evaluating EBC biomarkers—including pH and H_2_O_2_—in horses referred for bronchoscopy due to respiratory symptoms found no significant correlations with TW or BAL cytology.^19^ Moreover, additional biomarkers should be considered in future studies, including nonvolatile organic compounds (non-VOCs) and metabolomic profiles. In a study by Bazzano et al.,^20^ a metabolomic approach applied to BAL and EBC fluids identified molecules such as myo-inositol and methanol as potential biomarkers for investigating asthma in horses. Additionally, recent studies have demonstrated the presence of extracellular vesicles in EBC, carrying microRNA cargo that may be useful for evaluating lung conditions; these should be considered in future equine EBC investigations.^21^

The present study had some limitations. One issue was sample loss, likely due to the limited cooling surface area of the collection device, highlighting the need for design improvements. Modifications to the equipment are already underway, guided by these initial findings. In previous studies, such as those by Whittaker et al.,^4^ Cathcart et al.,^22^ and Crowley et al.,^23^ exhaled breath condensate (EBC) was collected over a 5 min period at −78 °C, resulting in sample volumes of approximately 0.7 mL, 0.4–1.4 mL, and 1.5 mL, respectively. Other investigations reported longer collection times ranging from 10 to 15 min, with sample volumes between 1.1 and 3 mL at −20 °C, and 0.5 to 2 mL at 0 °C.^14,20^ While these studies show some consistency regarding collection times and yields, considerable variability remains, reinforcing the lack of standardized protocols for EBC sampling. Notably, none of the cited studies reported sample loss associated with the collection techniques employed. Moreover, contamination with saliva—an issue described in other EBC studies—did not occur with the equipment used in the present study, which may represent an additional advantage of the proposed design.

Another limitation was the relatively small number of animals included. However, the sample size was considered sufficient to assess the primary aim of this preliminary evaluation—namely, testing for potential intra- and interday variability, which was not observed. Although the inclusion criteria required clinically healthy animals with no history of respiratory disease, the sample ultimately included horses with both normal cytological profiles and profiles compatible with mild to moderate asthma, based on bronchoalveolar lavage (BAL) fluid analysis. Given the main objective of this study—evaluating the collection capacity and repeatability of the newly developed EBC device—analyses were performed on the combined group. Although separate analyses revealed no significant differences between healthy and asthmatic horses, the inclusion of both subgroups may have influenced the overall findings. Future studies with larger, well-defined groups based on established consensus criteria for equine asthma syndrome^13^ are necessary to expand upon these observations.

## 5 Conclusion

In the present study, using a new portable EBC collector, it was concluded that EBC can be collected at any and multiples time of the day without affecting pH and H_2_O_2_ results. The sample volume varied among the tested mares, which may be related to the intrinsic characteristics of the animals, climatic conditions, the protocol used, or the design of the proposed equipment.

## Supplementary Material



## Notes

Supplementary Tables 1 and 2 present the cellular composition, pH values, and hydrogen peroxide (H_2_O_2_) concentrations in bronchoalveolar lavage (BAL) fluid from horses classified as healthy or as having mild to moderate asthma, respectively. Supplementary Tables 3 and 4 provide the corresponding data for tracheal wash (TW) fluid from healthy and asthmatic horses (PDF)
