# Supplementary material for: Intraday and Interday Evaluation of pH and Hydrogen Peroxide in the Exhaled Breath Condensate of Horses Using A Portable Device
Source: ACS Omega. 2025 Oct 6;10(41):48412–7. doi: 10.1021/acsomega.5c05941 (PMC12547516; doi:10.1021/acsomega.5c05941)
Supplement: Supplementary file 1 [file ao5c05941_si_001.pdf]

Intraday and interday evaluation of pH and hydrogen peroxide in the exhaled breath condensate of horses using a portable device.

Bianca Barbosa [a], *Thasla F. Santi* [a], *Ana C. Rodak* [b], *Maria F. Nogara* [b], *Lidiane M.B. Leite* [c], *Saulo H. Weber* [a], *Cleber Niels* [a], *Ruan R. Daros* [a,d], *Pedro V. Michelotto* [a,d]*

[a] Graduate Program in Animal Science, Pontifícia Universidade Católica Paraná, Rua Imaculada Conceição 1155, Prado Velho, 80215-901, Curitiba PR, Brazil

[b] Veterinary Medicine, Pontifícia Universidade Católica Paraná, Rua Imaculada Conceição 1155, Prado Velho, 80215-901, Curitiba PR, Brazil

[c] Core for Cell Technology, School of Medicine and Life Sciences, Pontifícia Universidade Católica do Paraná, Rua Imaculada Conceição 1155, Prado Velho, 80215-901, Curitiba PR, Brazil.

[d] EthoLab – Animal Welfare and Applied Ethology Lab, Rua Imaculada Conceição 1155, Prado Velho, 80215-901, Curitiba PR, Brazil, Pontifícia Universidade Católica do Paraná.

Keywords: Diagnosis, Equine, Lung disease, Respiratory, Sustainability.

Bronchoalveolar lavage fluid (BALF)

Table S1 – This table summarizes the cellular composition, pH values, and hydrogen peroxide (H_2O_2) concentrations measured in the bronchoalveolar lavage (BAL) fluid of horses classified as healthy based on clinical and cytological criteria.

Horse	Eosinoph	Macroph	Neutroph	Lymphoc	Mast Cell	pH A	pH B	pH C	pH D	pH E	H2O2 A	H2O2 B	H2O2 C	H2O2 D	H2O2 E
1	0.0	77.0	1.3	21.7	0.0	7.3	6.5	6.2	6.6	NP	0.741	1.360	NP	1.033	1.768
4	0.0	43.3	1.3	55.3	0.0	8.2	7.6	7.5	NP	7.6	2.077	NP	1.536	2.010	0.984
7	0.0	74.7	1.7	23.7	0.0	7.7	7.4	7.7	7.6	7.5	0.808	0.722	1.154	0.650	0.632
9	0.0	55.3	0.3	44.3	0.0	8.4	7.7	8.5	8.4	NP	0.313	0.478	NP	1.087	1.016
11	1.0	53.3	2.7	43.0	0.0	8.3	7.5	7.8	8.2	NP	0.737	1.421	NP	1.351	0.524
**Mean**	**0.2**	**60.7**	**1.5**	**37.6**	**0.0**	**8.0**	**7.3**	**7.5**	**7.7**	**7.6**	**0.935**	**0.995**	**1.345**	**1.226**	**0.985**

A, B and C are samples collected at the same day at 8 AM, 12 PM and 4 PM; D and E are samples collected at 8 AM in two consecutive days. NP. not possible.

Table S2 – This table summarizes the cellular composition, pH values, and hydrogen peroxide (H_2O_2) concentrations measured in the bronchoalveolar lavage (BAL) fluid of horses classified as mild to moderate asthma based on cytological criteria.

Horse	Eosinoph	Macroph	Neutroph	Lymphoc	Mast Cell	pH A	pH B	pH C	pH D	pH E	H2O2 A	H2O2 B	H2O2 C	H2O2 D	H2O2 E
2	1.7	45.0	14.0	39.3	0.0	7.0	NP	6.9	NP	NP	0.272	NP	NP	NP	0.387
3	1.7	44.7	32.7	20.7	0.3	NP	NP	NP	NP	NP	NP	NP	NP	NP	NP
5	0.3	18.0	20.0	61.7	0.0	NP	NP	NP	NP	NP	1.101	NP	NP	NP	NP
6	0.0	37.7	27.0	35.0	0.3	7.9	8.6	8.4	8.4	8.7	0.736	1.336	0.792	0.868	0.701
8	0.0	53.7	12.7	33.7	0.0	NP	7.7	8.2	7.3	7.0	NP	0.202	0.416	0.545	1.847
10	0.0	25.3	7.7	67.0	0.0	8.5	8.2	7.6	8.4	NP	1.885	0.690	NP	0.967	1.381
**Mean**	**0.6**	**37.4**	**19.0**	**42.9**	**0.1**	**7.8**	**8.2**	**7.8**	**8.0**	**7.0**	**0.999**	**0.743**	**0.604**	**0.793**	**1.079**

A, B and C are samples collected at the same day at 8 AM, 12 PM and 4 PM; D and E are samples collected at 8 AM in two consecutive days. NP. not possible.

Tracheal Wash (TW) –

Table S3 – This table summarizes the cellular composition, pH values, and hydrogen peroxide (H_2O_2) concentrations measured in the tracheal wash (TW) fluid of horses classified as healthy based on clinical and cytological criteria.

Horse	Eosinoph	Macroph	Neutroph	Lymphoc	Mast Cell	pH A	pH B	pH C	pH D	pH E	H2O2 A	H2O2 B	H2O2 C	H2O2 D	H2O2 E
2	0.3	52.3	19.3	28.0	0.0	7.0	NP	6.9	NP	NP	0.272	NP	NP	NP	0.387
3	0.0	75.7	13.7	10.7	0.0	NP	NP	NP	NP	NP	NP	NP	NP	NP	NP
4	1.0	60.3	1.3	37.3	0.0	8.2	7.6	7.5	NP	7.6	2.077	NP	1.536	2.010	0.984
7	0.0	60.0	1.7	38.3	0.0	7.7	7.4	7.7	7.6	7.5	0.808	0.722	1.154	0.650	0.632
8	0.0	74.7	16.0	9.3	0.0	NP	7.7	8.2	7.3	7.0	NP	0.202	0.416	0.545	1.847
9	1.0	57.7	0.7	40.7	0.0	8.4	7.7	8.5	8.4	NP	0.313	0.478	NP	1.087	1.016
11	0.3	60.3	2.7	36.7	0.0	8.3	7.5	7.8	8.2	NP	0.737	1.421	NP	1.351	0.524
**Mean**	**0.4**	**63.0**	**7.9**	**28.7**	**0.0**	**7.9**	**7.6**	**7.8**	**7.9**	**7.4**	**0.842**	**0.706**	**1.035**	**1.129**	**0.898**

A, B and C are samples collected at the same day at 8 AM, 12 PM and 4 PM; D and E are samples collected at 8 AM in two consecutive days. NP. not possible.

Table S4 – This table summarizes the cellular composition, pH values, and hydrogen peroxide (H_2O_2) concentrations measured in the tracheal wash (TW) fluid of horses classified as asthmatics based on cytological criteria.

Horse	Eosinoph	Macroph	Neutroph	Lymphoc	Mast Cell	pH A	pH B	pH C	pH D	pH E	H2O2 A	H2O2 B	H2O2 C	H2O2 D	H2O2 E
1	1.3	57.7	21.3	19.7	0.0	7.3	6.5	6.2	6.6	NP	0.741	1.360	NP	1.033	1.768
5	0.0	16.3	48.0	35.7	0.0	NP	NP	NP	NP	NP	1.101	NP	NP	NP	NP
6	0.0	23.3	39.7	37.0	0.0	7.9	8.6	8.4	8.4	8.7	0.736	1.336	0.792	0.868	0.701
10	0.0	19.0	47.7	33.3	0.0	8.5	8.2	7.6	8.4	NP	1.885	0.690	NP	0.967	1.381
**Mean**	**0.3**	**29.1**	**39.2**	**31.4**	**0.0**	**7.9**	**7.8**	**7.4**	**7.8**	**8.7**	**1.116**	**1.128**	**0.792**	**0.956**	**1.283**

A, B and C are samples collected at the same day at 8 AM, 12 PM and 4 PM; D and E are samples collected at 8 AM in two consecutive days. NP. not possible.
